# Experimental infection of lambs with tick-borne encephalitis virus and co-infection with *Anaplasma phagocytophilum*

**DOI:** 10.1371/journal.pone.0226836

**Published:** 2019-12-19

**Authors:** Katrine M. Paulsen, Erik G. Granquist, Wenche Okstad, Rose Vikse, Karin Stiasny, Åshild K. Andreassen, Snorre Stuen

**Affiliations:** 1 Department of Virology, Division for Infection Control and Environmental Health, Norwegian Institute of Public Health, Oslo, Norway; 2 Department of Production Animal Clinical Sciences, Faculty of Veterinary Medicine, Norwegian University of Life Sciences, Oslo, Norway; 3 Section of Small Ruminant Research and Herd Health, Department of Production Animal Clinical Sciences, Faculty of Veterinary Medicine, Norwegian University of Life Sciences, Sandnes, Norway; 4 Center for Virology, Medical University of Vienna, Vienna, Austria; University of Padua, ITALY

## Abstract

Tick-borne encephalitis virus (TBEV) is a zoonotic pathogen which may cause tick-borne encephalitis (TBE) in humans and animals. More than 10,000 cases of TBE are reported annually in Europe and Asia. However, the knowledge on TBE in animals is limited. Co-infection with *Anaplasma phagocytophilum* and louping ill virus (LIV), a close relative to TBEV, in sheep has been found to cause more severe disease than single LIV or *A*. *phagocytophilum* infection. The aim of this study was to investigate TBEV infection and co-infection of TBEV and *A*. *phagocytophilum* in lambs. A total of 30 lambs, aged five to six months, were used. The experiment was divided into two. In part one, pre- and post-infection of TBEV and *A*. *phagocytophilum* was investigated (group 1 to 4), while in part two, co-infection of TBEV and *A*. *phagocytophilum* was investigated (group 5 and 6). Blood samples were drawn, and rectal temperature was measured daily. Lambs inoculated with TBEV displayed no clinical symptoms, but had a short or non-detectable viremia by reverse transcription real-time PCR. All lambs inoculated with TBEV developed neutralizing TBEV antibodies. Our study is in accordance with previous studies, and indicates that TBEV rarely causes symptomatic disease in ruminants. All lambs inoculated with *A*. *phagocytophilum* developed fever and clinical symptoms of tick-borne fever, and *A*. *phagocytophilum* was present in the blood samples of all infected lambs, shown by qPCR. Significantly higher mean TBEV titer was detected in the group co-infected with TBEV and *A*. *phagocytophilum*, compared to the groups pre- or post-infected with *A*. *phagocytophilum*. These results indicate that co-infection with TBEV and *A*. *phagocytophilum* in sheep stimulates an increased TBEV antibody response.

## Introduction

The disease tick-borne encephalitis (TBE) in humans and animals is caused by tick-borne encephalitis virus (TBEV). TBEV is a member of the genus *flavivirus* within the family *flaviviridae*, and it is mainly transmitted to humans and animals through bites by TBEV-infected *Ixodes ricinus* or *Ixodes persulcatus* ticks [1]. In addition, TBEV has been detected in unpasteurized milk from domestic ruminants and there are reported human cases of alimentary TBE from consumption of unpasteurized milk and other dairy products [2–9].

In humans, TBE may vary from asymptomatic to severe infection in the central nervous system, and the number of annually reported human TBE cases is increasing in Europe and Asia [10, 11]. Most animals do not develop symptomatic disease when infected with TBEV. However, the knowledge on TBE in animals is limited. TBE has been described with neurological symptoms in dogs, horses, and, in one case, monkey (*Macaca sylvanus*) [12–16]. TBE in small ruminants is presumably rare, with only a few reported cases [17, 18]. Large and small mammals along with migratory birds are known to be important for the distribution and transmission of the virus [19–26].

*Anaplasma phagocytophilum* is the causative agent of tick-borne fever in ruminants and is transmitted by the same tick species as TBEV in Europe, namely *I*. *ricinus* [27]. The intracellular bacterium is known to affect domestic ruminants, humans and wild animals [27, 28]. *A*. *phagocytophilum* has a great negative impact on the sheep farming and it has been estimated that more than 300,000 lambs are infected by *A*. *phagocytophilum* annually in Norway [29]. Infection with *A*. *phagocytophilum* results in immune suppression and the most typical symptoms in domestic ruminants include high fever, depression, reduced appetite, and sudden drop in milk yield [30, 31]. Reduced weight gain in infected lambs has also been observed [32, 33].

Because several tick-borne pathogens often circulate in the same area, humans and animals may be infected with multiple pathogens from tick-bites [34]. A recent study in Norway by Kjelland et al. (2018), reported co-infected ticks with *Borrelia afzelii* and *Neoehrlichia mikurensis*. The same study found several tick-borne pathogens, including TBEV and *A*. *phagocytophilum*, in the same locations [35]. Co-infection with *A*. *phagocytophilum* and other pathogens in sheep has been found to cause more severe disease compared to infection with a single pathogen [36, 37]. Previous studies have shown that co-infection with *A*. *phagocytophilum* and louping ill virus (LIV) in sheep may give fatal clinical outcomes [36, 38]. TBEV and LIV are closely related, and it has been speculated whether similar clinical outcomes could occur from co-infection with *A*. *phagocytophilum* and TBEV. A recently published experimental study on the immune responses to TBEV and LIV in sheep, showed that the infected sheep developed neutralizing antibodies for both viruses, which seemed to limit the infection caused by TBEV, but not the infection caused by LIV [39]. Furthermore, prior inoculation with TBEV appeared to reduce the disease severity and viremia caused by LIV, but it did not prevent LIV infection [39]. The objective of this study was to study the effect of TBEV infection and co-infection of TBEV and *A*. *phagocytophilum* in lambs.

## Materials and methods

### Ethics statement

The study was authorized by the Norwegian Animal Research Authority (Norwegian Food Safety Authority, FOTS ID 8632, FOTS ID 8135). Blood samples were collected by trained veterinarians, and all lambs were observed daily.

### Experimental design and blood sampling

This study was conducted at the Norwegian University of Life Sciences (NMBU) in Sandnes, Norway. The study was divided in two parts. A total of 30 lambs, at the age of five to six months of the breed “Norwegian white sheep”, were used. Part one included only rams, and was performed in the autumn of 2017. Part two consisted entirely of ewes, and was carried out in the autumn of 2018 (Table 1).

**Table 1:** Overview of the study groups and the experimental design of part one and part two of the experimental study with infection of tick-borne encephalitis virus (TBEV) and *Anaplasma phagocytophilum* in lambs.

Part one: Pre- and post- infection of TBEV and *A*. *phagocytophilum*					Part two: Co-infection of TBEV and *A*. *phagocytophilum*	
* *						
Day	Group 1_a_	Group 2_a_	Group 3_a_	Group 4_a_	Group 5_a_	Group 6_a_
0	Inoculated with TBEV_b_	Inoculated with *A*. *phagocytophilum*_*c*_	Inoculated with *A*. *phagocytophilum*_c_	Negative controls. Inoculated with uninfected cell medium_d_	Inoculated with TBEV and *A*. *phagocytophilum*_*b*__,__*c*_	Negative controls. Inoculated with physiological saline solution_d_
21	Inoculated with *A*. *phagocytophilum*_*c*_	Inoculated with uninfected cell medium_c_	Inoculated with TBEV_b_	Negative controls. Inoculated with uninfected cell medium_d_	End of experiment	
42	End of experiment				-	

^a^ Each group consisted of five lambs.

^b^TBEV was inoculated subcutaneously (1 ml of the strain Hohosterwitz, approximately 6.5x10^6^ focus forming units per ml (FFU/ml).

^*c*^*A*. *phagocytophilum* was inoculated intravenously (0.4 ml of heparinised sheep blood stabilized with 10% demethyl sulphoxide (DMSO), approximately 1x10^6^ infected cells, GenBank accession number M73220).

^d^1 ml negative control medium and saline were inoculated subcutaneously.

The main reason for the difference in gender between part one and part two was the limited number of animals available. No differences between genders have been observed previously in experimental infection with *A*. *phagocytophilum* in sheep [40]. The main reason to split male and female lambs in two separate groups was to avoid disturbances due to rutting behavior of young males. The lambs were used to handling before the start of the experiment. Sedatives were not used.

In part one, the animals were divided into four groups of five ram lambs (group 1–4, Table 1). On day 0, lambs in group 1 were inoculated with 1 ml of the TBEV-strain Hochosterwitz (European subtype, approximately 6.5x10^6^ focus forming units per ml (FFU/ml)), and lambs in group 2 and 3 were inoculated with 1 ml *A*. *phagocytophilum* (0.4 ml of heparinised sheep blood stabilized with 10% demethyl sulphoxide (DMSO), approximately 10^6^ infected cells, GenBank accession number M73220). The lambs in group 4 were negative controls, and were inoculated with uninfected cell medium from the virus cultivation. On day 21, lambs in group 1 were inoculated with the same strain of *A*. *phagocytophilum*, and lambs in group 3 with the same strain of TBEV as described above. Lambs in group 2 served as *A*. *phagocytophilum* controls.

TBEV and the negative control medium were inoculated subcutaneously and *A*. *phagocytophilum* intravenously. The experimental infection model with intravenous inoculation of *A*. *phagocytophilum* has been used for several years at NMBU in Sandnes [40]. In addition, no difference in clinical manifestation has previously been observed after subcutaneous, intradermal or intravenous inoculation, except for a delay in incubation period after subcutaneous/intradermal inoculation. TBEV was inoculated subcutaneously to mimic tick bites, and because TBEV has been inoculated subcutaneously in mouse models and in studies in sheep previously. For practical reasons and to avoid any mixture with the subcutaneous TBEV inoculation, *Anaplasma phagocytophilum* was inoculated intravenously.

Blood samples were drawn from *Vena jugularis* using vacuette tubes from all lambs on day 0, 2, 4, 6, 8, 10, 14, 18, 21, 23, 25, 27, 29, 31, 35, 39 and 42 (two EDTA tubes of 2 ml and one serum-tube with clot activator of 9 ml, Vacuette^®^ Greiner Bio-One GmbH, Kremsmünster, Austria). The experimental period in part one ended on day 42 (Table 1). All lambs from part one of the study were euthanized, and brain samples were obtained for PCR analysis. The animals were euthanized by intravenous injection of pentobarbital sodium 400 mg/ml (Euthasol vet, Le Vet B.V., Oudewater, The Netherlands) at 140 mg/kg).

Study part two was designed similarly with two groups of five ewe lambs each (group 5 and 6 Table 1). The experimental period in part two ended on day 21. The same strains and batches of TBEV and *A*. *phagocytophilum* as above were inoculated to group 5 on day 0, while physiological saline solution was used as negative control and inoculated to group 6 on the same day. Blood samples were drawn from *Vena jugularis* using vacuette tubes all animals on day 0, 2, 4, 6, 8, 10, 14, 18 and 21 (two 2 ml EDTA-tubes, and one 4 ml serum-tube with clot activator, Vacuette^®^ Greiner Bio-One GmbH, Kremsmünster, Austria).

All serum tubes were separated by centrifugation within two hours post sampling, and stored at -80 ^o^C until analysis. One EDTA tube was stored at -20 ^o^C for *A*. *phagocytophilum* PCR, while the second tube was used for hematology.

### Hematology

Hematological analyses were performed on the ADVIA 120 instrument (Siemens healthcare, Erlangen Germany) with veterinary software for sheep blood.

### Detection of tick-borne encephalitis virus

RNA from the serum samples was extracted on QIAcube with QIAamp^®^ Viral RNA mini kit (QIAGEN GmbH, Hilden, Germany) according to the manufacturer’s recommendations. RNA from the brain samples was extracted by RNeasy mini kit (QIAGEN GmbH, Hilden, Germany) according to the manufacturer’s recommendations. Immediately after the extraction process, the RNA was reversely transcribed to cDNA with random primers (High-Capacity cDNA Reverse Transcription Kit, Applied Biosystems, Foster city, CA, USA). To detect TBEV RNA, an in-house reverse transcriptase (RT) real-time PCR was performed according to Andreassen et al. (2012). The real-time PCR amplifies a 54 base pair (bp) fragment located on the envelope gene of TBEV. A positive RNA control (“Soukup”) was used in the real-time PCR [41]. Nuclease free water was used as negative control.

### Detection of antibodies to tick-borne encephalitis virus

Serum samples from lambs were analyzed for TBEV IgG by a commercial enzyme linked immunosorbent assay (ELISA, Enzygnost^®^ Anti-TBE virus IgG, Siemens Healthcare, GmbH, Marburg, Germany) according to the manufacturer’s protocol, with one modification: the conjugate was changed to Peroxidase-Labeled Anti-Sheep IgG antibody (KPL, Gaithersburg, USA). The IgG conjugate was diluted 1:50,000. Serum from sheep vaccinated against TBEV with the TicoVac-vaccine (Pfizer Ltd, Ramsgate Road, Sandwich, Kent, CT13 9NJ, UK) was used as positive control, and serum from sheep which had never been exposed to ticks was used as negative control [8]. All positive and borderline samples from the ELISA were further tested in a TBEV-specific serum neutralization test (SNT) at the Center for Virology of the Medical University of Vienna, as described previously [42].

### Detection of *Anaplasma phagocytophilum*

DNA from the EDTA blood samples were extracted on MagNA Pure 96 with MagNA Pure 96 DNA and viral NA large volume kit (Roche Molecular Systems, Inc. Basel, Switzerland) according to the manufacturer’s recommendations. To detect *A*. *phagocytophilum* DNA, a quantitative real-time PCR method was performed according to Henningsson et al. 2015. This method amplifies a 64 bp fragment of the gltA gene of the bacterium [43]. A positive *A*. *phagocytophilum* control and a synthetic plasmid (pAP-GltA cloned in pUC57, GenScript Cooperation, Scotch plains, NJ) were used in the qPCR. Nuclease free water was used as negative control.

### Statistics

All clinical and laboratory data were collected in Microsoft Excel (2016) spreadsheets and transferred to Stata 14.2 for Windows (StataCorp, 4905 Lakeway Drive. College Station, Texas 77845) for statistical analysis. The quality of data and distributions were analyzed using tabulations and histograms. Initial analyses included multilevel linear regression modelling of each of the continuous outcome variables; rectal temperature, neutrophil counts, lymphocyte counts, monocyte counts, quantitative PCR of *A*. *phagocytophilum* and TBEV titer. Predictors were “Group” (exposure) and “Day” of infection and the random effects variable was “The individual lambs”. The statistical analyses were performed on day 0 to day 21 post inoculation with TBEV and *A*. *phagocytophilum*. Residuals were estimated and visualized in quantile plots. p <0.05 was considered significant. Additional, descriptive statistical analyses were performed in Excel and GraphPad Prism version 8.0.0 for Windows (GraphPad Software, San Diego, California USA).

## Results

### Part one: pre- and post-infection of TBEV and *A*. *phagocytophilum*

The lambs in group 1 and 3, which had been inoculated with TBEV, displayed no clinical TBE symptoms or fever, and had a short or non-detectable viremia by RT real-time PCR on serum samples. On day two post TBEV infection, four of five lambs in group 1 tested positive for TBEV in the serum, while in group 3 two of five lambs were positive. One of five lambs in group 3 tested positive for TBEV on day four post TBEV infection. All samples were negative on day six and throughout the experiment (S1 Table). The brain samples collected from the lambs at the end of the experiment (day 42) were all found to be TBEV negative by RT real-time PCR (data not shown).

**Fig 1:**
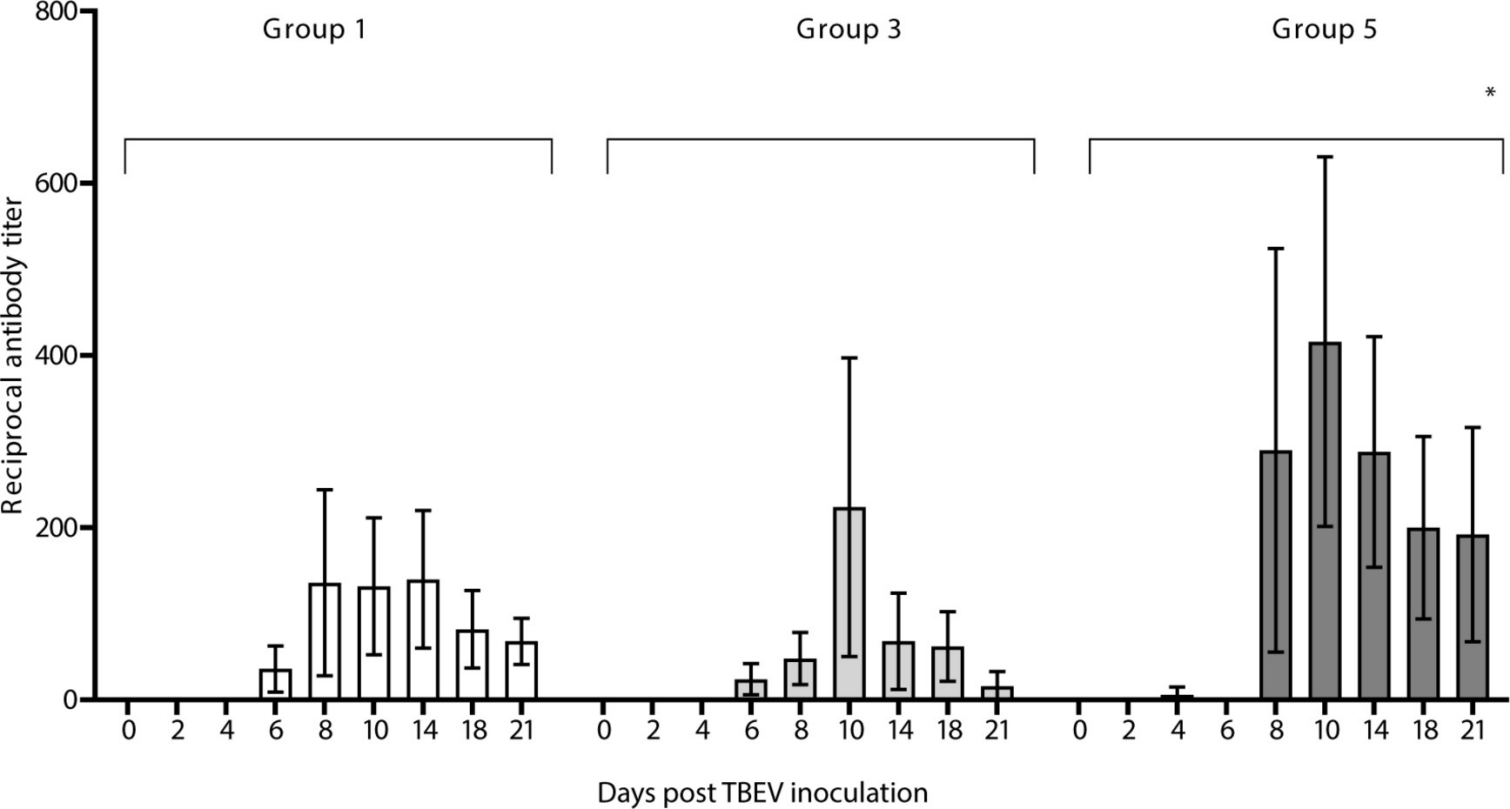
Mean reciprocal TBEV antibody titer in lambs post TBEV infection. TBEV titers (Y axis) were measured by serum neutralization test in group 1, 3 and 5 on day 0 to day 21 post inoculation with TBEV (X axis). Group 5 had significantly higher mean TBEV titer values than group 1 and 3, indicated with*. Standard deviations (SD) are illustrated with error bars. The groups which were not inoculated with TBEV are not included in the figure, and did not develop neutralizing antibodies to the virus.

The results from serum neutralization test showed that the lambs inoculated with TBEV (group 1 and 3) developed neutralizing antibodies to the virus (Fig 1, S1 Table). The lambs had detectable neutralizing antibodies in the serum from day six post TBEV infection, and throughout the experiment. No significant difference in the mean TBEV titer between group 1 and 3 was found (p>0.05).

**Fig 2:**
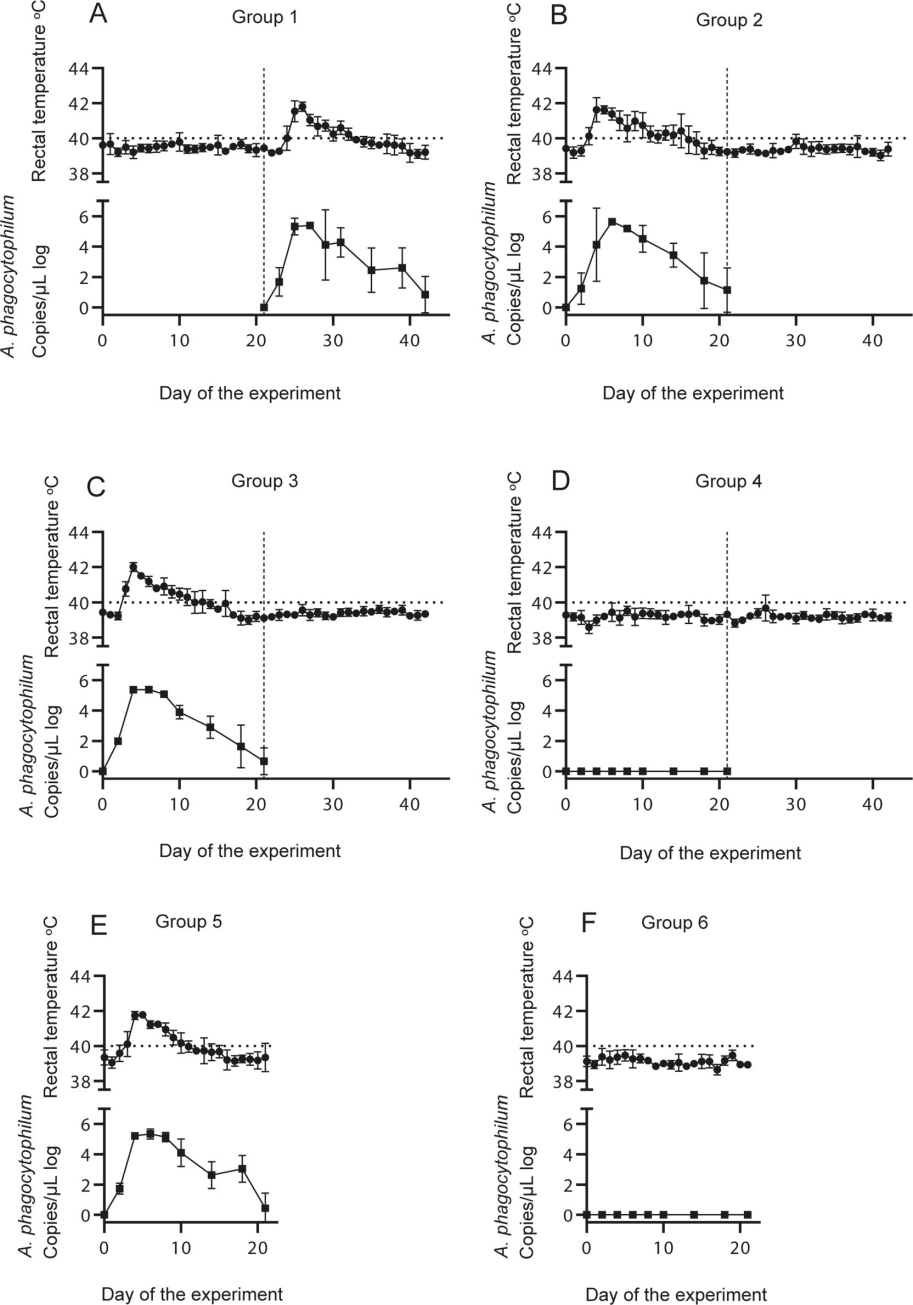
Mean rectal temperature and mean concentration of *Anaplasma phagocytophilum* in the lambs. The mean rectal temperature (Y axis) and mean concentration of *A*. *phagocytophilum* in the blood measured by qPCR (copies per μL (logarithmic), Y axis) of each group on day 0 to day 42 post inoculation (X axis). The dotted line at 40 degrees Celsius indicates fever. The vertical dashed line in graph A to D indicates the second challenge day on day 21. Part two of the experiment (graph E and F) concluded on day 21. Standard deviations (SD) are illustrated by the error bars.

All lambs inoculated with *A*. *phagocytophilum* (group 1, 2 and 3) developed fever and clinical symptoms of *A*. *phagocytophilum* infection (Fig 2, S1 Table). One of the lambs was diagnosed with pneumonia and was euthanized before the end of the study according to animal welfare standards. *A*. *phagocytophilum* was detected by qPCR in all blood samples from day 2 post infection and throughout the experiment (Fig 2, S1 Table). No significant difference in the mean *A*. *phagocytophilum* concentration between group 1, 2 and 3 was found (p>0.05). Furthermore, no significant difference in the mean rectal temperature related to the *A*. *phagocytophilum* infection between group 1, 2 and 3 was found (p>0.05).

**Fig 3:**
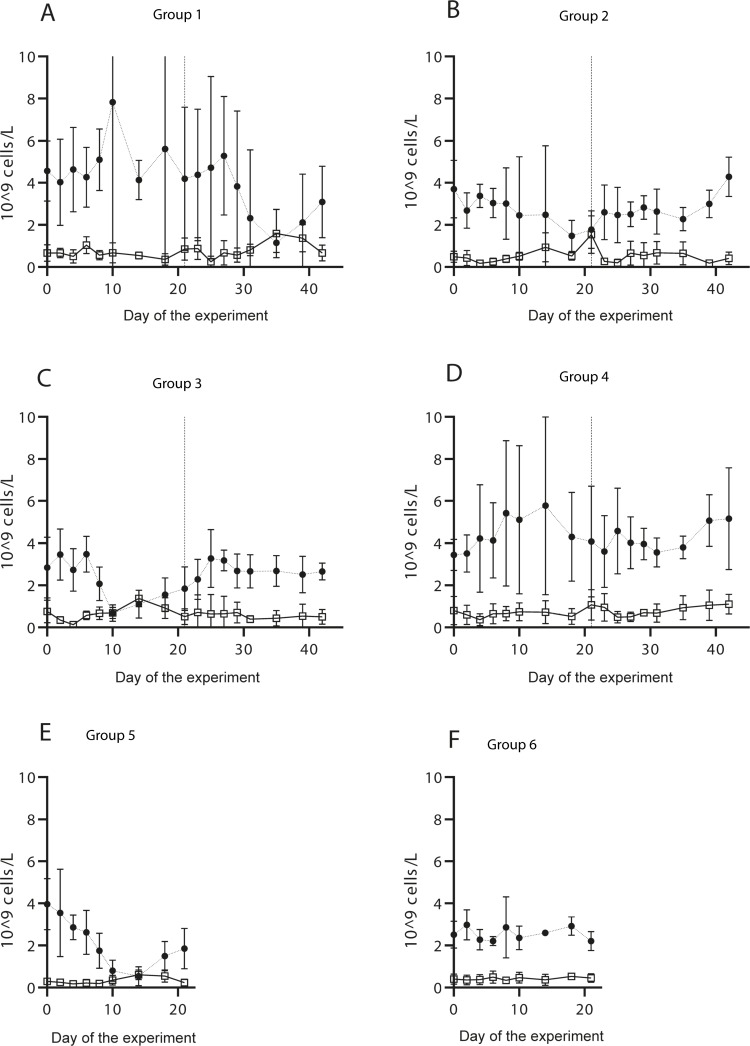
Mean counts of neutrophils and monocytes in the lambs in the experimental study. Mean counts (Y axis) of neutrophils (circular dots) and monocytes (squares) in the experiment. Normal counts in sheep are 0.8–5.0 (10^9^ cells per liter) for neutrophils and <0.75 (10^9^ cells per liter) for monocytes. The vertical dashed line in graph A to D indicates the second challenge on day 21. Standard deviations (SD) are illustrated by the error bars. The lack of endpoints in some error bars in graph A are due to outliers.

For the hematological analysis, group 2 had a significantly higher mean monocyte count compared to group 1 and 3 (p <0.05). No significant difference in the mean neutrophil and lymphocyte counts was found between group 1, 2 and 3 (p>0.05, Fig 3, S1 Table).

### Part two: Co-infection of TBEV and *A*. *phagocytophilum*

The lambs in group 5, which were co-infected with TBEV and *A*. *phagocytophilum*, displayed no clinical TBE symptoms, and the viremia was either not detectable or short-lived. Two of five lambs had detectable TBEV RNA in serum on day two, and one of five on day four and six. All serum samples tested negative for TBEV RNA on day eight and throughout the experiment. Similarly to part one in the present study, all lambs inoculated with TBEV developed neutralizing TBEV antibodies from day 4 and 8 post inoculation (Fig 1, S1 Table).

The lambs in group 5 developed fever and clinical signs of tick-borne fever, and the bacterium was detected by qPCR from day 2 post infection and throughout the experiment (Fig 2, S1 Table).

### Statistical comparison of study part one and two

A significantly higher mean TBEV titer was found in group 5 where the lambs were co-infected with TBEV and *A*. *phagocytophilum*, compared to group 1 which received an infection of TBEV on day 0 and *A*. *phagocytophilum* on day 21 (p<0.05). Similarly, group 5 had a significantly higher mean TBEV titer compared to group 3 which had been infected with *A*. *phagocytophilum* on day 0 and TBEV on day 21 (p<0.05). No differences in terms of viremia between pre-, post- and co-infection of TBEV and *A*. *phagocytophilum* was found.

No significant difference was found in the mean rectal temperature related to the *A*. *phagocytophilum* infection or the mean concentration of *A*. *phagocytophilum* in the blood samples between group 1, 2, 3 and 5 (p>0.05).

For the hematological analysis, a significantly higher mean count of monocytes was found after inoculation with *A*. *phagocytophilum* in group 1 (day 21–42) and group 3 (day 0–21) compared to group 5 (day 0–21). Similarly, post TBEV inoculation, a significantly higher mean number of monocytes was found in group 1 (day 0–21) than in group 5 (day 0–21). No significant difference in the mean count of neutrophils and lymphocytes was found between the *A*. *phagocytophilum* infected groups in part 1 and part 2, however, a significantly higher mean neutrophil count was found in group 1 compared to group 5 on day 0 to 21 post TBEV-inoculation (p<0.05, S1 Table).

## Discussion

There is a lack of information on the veterinary aspects of TBEV. This study aimed to investigate infection of TBEV and co-infection of TBEV and *A*. *phagocytophilum* in lambs. All TBEV infected lambs developed neutralizing TBEV antibodies, without displaying any clinical symptoms of TBE, and had a very short viremia. A significantly higher mean TBEV titer was found in the group co-infected with TBEV and *A*. *phagocytophilum* compared to the other groups. These results indicate that co-infection of TBEV and *A*. *phagocytophilum* in lambs may stimulate a higher TBEV antibody response compared to a single infection of TBEV, or a prior infection with *A*. *phagocytophilum*. The reason for this is, however, unknown.

The significant difference in the TBEV antibody titer could have been affected by the difference of the gender of the lambs in part one (rams) and part two (ewes). A previous study on *A*. *phagocytophilum* infection in laboratory mice found that infected male mice had increased *A*. *phagocytophilum* DNA load and number of infected neutrophils [44]. In the present study, no significant difference was found in the mean *A*. *phagocytophilum* DNA load, but a significantly higher mean neutrophil count was found in group 1 compared to group 5 post TBEV infection. Although TBEV viremia was low or non-detectable, the differences in gender could have affected the TBEV titers and the neutrophil counts. TBEV infection of lambs from different genders and ages have, however, not shown any differences in the clinical symptoms (unpublished data).

In our study, the mean number of monocytes was found to be significantly higher in group 2 than in all the other groups infected with *A*. *phagocytophilum*. Furthermore, groups 1 and 3 had a significantly higher mean monocyte count compared to group 5. Monocytes have been found to be important in combating *A*. *phagocytophilum* infection [45], and also to contribute to the cell-mediated immune response to TBEV [46, 47]. A significantly higher mean monocyte count was found in group 1 (day 0 to 21) than in group 5 (day 0 to 21) post TBEV infection. These results may indicate that when a single infection of *A*. *phagocytophilum* or TBEV occur (group 1 and 3), a higher cell-mediated immune response is developed, compared to co-infection with TBEV and *A*. *phagocytophilum* (group 5). However, no significant differences were found in the mean bacterial load of *A*. *phagocytophilum*, nor the clinical symptoms of the lambs.

The results from our study are in accordance with previous studies and together they indicate that TBEV rarely leads to symptomatic disease in sheep [17, 18, 39]. Co-infection with LIV and *A*. *phagocytophilum* is known to cause severe disease in sheep [36]. In our study, co-infection with *A*. *phagocytophilum* and TBEV did not seem to impact the clinical symptoms in lambs, even though LIV is genetically closely related to TBEV [48]. The absence of clinical TBE cases in sheep may be due to poor replication of the virus in sheep cells [39].

A recent experimental study on TBEV and LIV in sheep by Mansfield et al. (2016), found no clinical symptoms following TBEV infection, although a neutralizing antibody response was established [39]. Similar results were found in the current study. The study by Mansfield et al. (2016), found that the low antibody titer post TBEV infection was likely a reflection of the low viral load within the sheep infected with TBEV. Comparable results were found in this study, where a low viremia was detected in some of the lambs a few days post TBEV inoculation. Although a low and short-lived viremia was found, there is a known possibility of alimentary transmitted TBEV, which shows that ruminants develop a viremia post TBEV infection [2–5, 49–52]. Furthermore, an experimental study in goats detected TBEV viremia with a duration of up to 19 days [53]. The reason for the prolonged viremic period detected in goats compared to sheep is unknown, but it might indicate that goats are more susceptible to TBEV infection than sheep, or that there are differences in the pathogenicity of the viral strains.

In summary, the present study shows that all TBEV-infected lambs developed neutralizing TBEV antibodies without displaying any clinical symptoms of TBE. A significantly higher mean TBEV titer was found in the group co-infected with TBEV and *A*. *phagocytophilum* compared to the other groups. For future experimental studies in domestic ruminants other and possibly more virulent TBEV-strains should be considered to confirm the effects of co-infection using animals of the same gender.

## Supporting information

S1 Table Data on the rectal temperature, hematological variables, PCR results of tick-borne encephalitis virus and *Anaplasma phagocytophilum*, enzyme-linked immunosorbent assay and TBE titers. Abbreviations in S1 Table:
TBEV PCR: Tick-borne encephalitis virus real-time polymerase chain reaction (0 = negative, 1 = positive)
TBEV ELISA: Tick-borne encephalitis enzyme-linked immunosorbent assay (0 = negative, 1 = positive)
TBEV titer: Tick-borne encephalitis titer by serum neutralization test
Wbc: White blood cells
Rbs: Red blood cells
Hgb: Hemoglobin
Hct: Hematocrit
MCV: Mean cell volume
MCH: Mean cellular hemoglobin
RDV: Red cell distribution width
HDW: Hemoglobin distribution width
Neut: Neutrophils
Lymp: Lymphocytes
Mono: Monocytes
Eos: Eosinophils
Baso: Basophils
LUC: Large unstained cells.
(XLSX)
